# Employment status at transplant influences ethnic disparities in outcomes after deceased donor kidney transplantation

**DOI:** 10.1186/s12882-021-02631-4

**Published:** 2022-01-03

**Authors:** Jasmin Divers, Sumit Mohan, W. Mark Brown, Stephen O. Pastan, Ajay K. Israni, Robert S. Gaston, Robert Bray, Shahidul Islam, Natalia V. Sakhovskaya, Alejandra M. Mena-Gutierrez, Amber M. Reeves-Daniel, Bruce A. Julian, Barry I. Freedman

**Affiliations:** 1 Division of Health Services Research, Department of Foundations of Medicine, NYU Long Island School of Medicine, Mineola, NY 11501 USA; 2 Winthrop Research Institute, NYU Long Island School of Medicine, Mineola, NY USA; 3 Division of Nephrology, Department of Medicine, Columbia University College of Physicians & Surgeons, New York, NY USA; 4 Department of Epidemiology, Mailman School of Public Health, Columbia University, NY, NY USA; 5 Department of Biostatistics and Data Sciences, Division of Public Health Sciences, Wake Forest School of Medicine, Winston-Salem, NC USA; 6 Renal Division, Department of Medicine, Emory University School of Medicine, Atlanta, GA USA; 7 Division of Nephrology, Department of Medicine, Hennepin Healthcare, University of Minnesota, Minneapolis, MN USA; 8 Minneapolis Medical Research Foundation, Minneapolis, MN USA; 9 University of Alabama at Birmingham School of Medicine, Birmingham, AL USA; 10 Department of Pathology & Lab Medicine, Emory School of Medicine, Atlanta, GA USA; 11 Department of Internal Medicine, Section on Nephrology, Wake Forest School of Medicine, Winston-Salem, NC USA

**Keywords:** Deceased donor kidney transplantation, Allograft failure, Kidney recipient mortality, Outcome disparity, Employment status

## Abstract

**Background:**

African American (AA) recipients of deceased-donor (DD) kidney transplants (KT) have shorter allograft survival than recipients of other ethnic groups. Reasons for this disparity encompass complex interactions between donors and recipients characteristics.

**Methods:**

Outcomes from 3872 AA and 19,719 European American (EA) DDs who had one kidney transplanted in an AA recipient and one in an EA recipient were analyzed. Four donor/recipient pair groups (DRP) were studied, AA/AA, AA/EA, EA/AA, and EA/EA. Survival random forests and Cox proportional hazard models were fitted to rank and evaluate modifying effects of DRP on variables associated with allograft survival. These analyses sought to identify factors contributing to the observed disparities in transplant outcomes among AA and EA DDKT recipients.

**Results:**

Transplant era, discharge serum creatinine, delayed graft function, and DRP were among the top predictors of allograft survival and mortality among DDKT recipients. Interaction effects between DRP with the kidney donor risk index and transplant era showed significant improvement in allograft survival over time in EA recipients. However, AA recipients appeared to have similar or poorer outcomes for DDKT performed after 2010 versus before 2001; allograft survival hazard ratios (95% CI) were 1.15 (0.74, 1.76) and 1.07 (0.8, 1.45) for AA/AA and EA/AA, compared to 0.62 (0.54, 0.71) and 0.5 (0.41, 0.62) for EA/EA and AA/EA DRP, respectively. Recipient mortality improved over time among all DRP, except unemployed AA/AAs. Relative to DDKT performed pre-2001, employed AA/AAs had HR = 0.37 (0.2, 0.69) versus 0.59 (0.31, 1.11) for unemployed AA/AA after 2010.

**Conclusion:**

Relative to DDKT performed before 2001, similar or worse overall DCAS was observed among AA/AAs, while EA/EAs experienced considerable improvement regardless of employment status, KDRI, and EPTS. AA recipients of an AA DDKT, especially if unemployed, had worse allograft survival and mortality and did not appear to benefit from advances in care over the past 20 years.

**Supplementary Information:**

The online version contains supplementary material available at 10.1186/s12882-021-02631-4.

## Background

Deceased donor (DD) kidney transplantation (KT) from African American (AA) donors is associated with shorter allograft survival compared to DDKT from donors of other races/ethnicities. Donor African ancestry is included as a risk factor in the calculation of the Kidney Donor Risk Index (KDRI), a measure of DD organ quality used to generate the Kidney Donor Profile Index in the US kidney allocation system [1, 2]. Similarly, AA recipients of DDKT have poorer outcomes, regardless of the race/ethnicity of the donor [3, 4].

Causes of ethnic differences in DDKT outcomes remain unclear; they are likely multifactorial, with inherited, environmental, and socioeconomic factors contributing to donor- and recipient-level effects. Several reports highlighted the adverse impact of genetics, poverty, geography, and lack of education on access to kidney transplantation and outcomes after engraftment [3, 5–10]. We demonstrated more rapid allograft failure after kidney transplantation from DDs with apolipoprotein L1 gene (*APOL1*) high-risk genotypes. We suggested that using *APOL1* genotyping instead of DD race might refine the KDRI by increasing the number of good quality kidneys for waitlisted recipients [11–15]. We and others reported genetic variants that affect AA DDKT outcomes either independently or through their interaction with *APOL1* kidney-risk variants [16–19]. Beyond *APOL1*, several biological factors independently contribute to, or interact with non-biological factors leading to poorer outcomes among AA DDKT recipients. For example, given fewer AA donors and greater allelic variation at the HLA locus, potential AA recipients are disadvantaged in an allocation system that includes HLA matching. Despite recognizing these limitations and related changes, AA wait longer for kidney transplantation, an important modifiable risk factor for adverse outcomes [20–22]. The situation is compounded by complex interactions between donor and recipient characteristics impacting long-term outcomes.

Herein, we attempt to measure the effects of recipient- and donor-specific factors and their interaction on observed racial/ethnic disparities by studying partner kidneys from DDs that are, by definition, genetically identical and were transplanted into recipients of different races. Analyses were restricted to AA and European American (EA) donors and recipients for ease of comparison. This approach provides better control for donor-level confounding factors, including donor-level genetic risk and race/ethnicity, on recipient outcomes after transplantation [1, 23].

## Methods

These analyses used donor and recipient data in the Scientific Registry of Transplant Recipients (SRTR) for kidneys procured and transplanted between October 1, 1987, and June 30, 2016. Analyses were restricted to AA or EA DDs who had both partnered kidneys transplanted, one to an AA recipient and the other to an EA recipient, yielding four groups of donor/recipient pairs (DRP): AA/AA, AA/EA, EA/AA, and EA/EA. This matched design better controlled for confounding by donor-related genetic, organ-specific, or socioeconomic factors and facilitated comparison of recipient-level factors contributing to observed racial disparities in outcomes. Donors or recipients < 18 years of age were excluded.

The primary outcome was death-censored time to kidney allograft failure, determined by the interval between transplantation dates and allograft loss. In patients with a functioning allograft, the final observation date was censored for death with function or at last follow-up before March 5th, 2016. A secondary outcome treating death as a competing risk (CR) was also considered. In this case, the final observation date was censored at death for individuals who died with a functioning allograft or at the most recent follow-up before March 5th, 2016, for living individuals with functioning allografts.

A split-half hypothesis-free analysis approach was applied where a random survival forest (RSF) model was fit in a randomly selected subset of the data representing 50% of the data to rank variables and their interaction with DRP based on their variable importance (VIMP) measure [24, 25]. RSF models implementing the conditional VIMP measures are robust to multicollinearity between predictors and are well-suited to detect interaction effects, which are of particular importance here [26, 27]. Analyses were repeated on the second half of the data and then on the complete data after observing strong reliability between the results obtained in the two subsets. Therefore, effect sizes and interaction effects with the DRP were estimated in the combined dataset using the top-ranked variables based on VIMP. This approach minimized the loss of statistical power caused by splitting the data into subsets [28]. Cox Proportional Hazard (CPH) models were fitted for death-censored allograft survival (DCAS) and the Fine and Gray model when death was considered a CR to allograft survival to obtain effect size estimates. The sandwich estimator was used to obtain a robust estimation of the covariance matrix associated with the parameter estimates to account for the correlation between allograft failure rate and time to failure of kidneys donated by a single individual to two recipients. Lin and Wei reported that this sandwich estimator was consistent and robust to several misspecifications of the Cox model [29]. Proportional hazard assumptions were checked by visual inspection of the log-log curve and assessing the Schoenfeld and martingale residuals [30]. Models were fitted separately following missing data imputation, which was performed within the RSF framework because RSF based-imputations have demonstrated high degree of robustness even in the presence of non-random missingness patterns [31, 32]. Ten imputed datasets were created, and the result obtained with these datasets were combined using established approaches [33–35]. Analyses were performed in SAS 9.4 and R 4.1. The RandomForestSCR package was used to fit Random Forest models for DCAS and the competing risk model [36].

## Results

The cohort consisted of 47,182 kidney transplants from 3872 AA and 19,719 EA DDs. Tables 1 and 2 display distributions of demographic variables and clinical characteristics for donors and recipients, respectively. Data are presented as median (Q_1_, Q_3_) for continuous and N (%) for categorical variables. All comparisons in these Tables were statistically significant (*p* < 0.0001).

**Table 1:** Demographic data for 23,591 deceased-donors (3872 African Americans and 19,719 European Americans)

Variable	All		AA donors		EA donors		***P***-value
	***N***	***Median (Q1, Q3), %***	***N***	***Median (Q1, Q3), %***	***N***	***Median (Q1, Q3), %***	
Female, %	23,591	40.0	3872	35.4	19,719	40.9	< 0.0001
Age, years	23,591	40.0 (27.0, 51.0)	3872	35.0 (24.0, 47.0)	19,719	41.0 (28.0, 51.0)	< 0.0001
BMI, kg/m^2^	20,869	25.7 (22.7, 29.8)	3529	25.8 (22.8, 30.1)	17,340	25.7 (22.7, 29.8)	0.05
Cardiac death, %	19,499	9.7	3343	4.1	16,156	10.8	< 0.0001
ECD, %	23,591	14.0	3872	12.0	19,719	14.4	< 0.0001
Hypertension, %	23,591	21.4	3872	26.8	19,719	20.3	< 0.0001
Kidney Donor Risk Index (KDRI)	19,395	1.3 (1.1, 1.7)	3326	1.3 (1.0, 1.6)	16,069	1.3 (1.1, 1.7)	< 0.0001
Serum creatinine, mg/dL	19,423	1.0 (0.7, 1.3)	3329	1.1 (0.9, 1.5)	16,094	0.9 (0.7, 1.2)	< 0.0001
Cold ischemia time, hours	22,327	16.0 (11.0, 22.9)	3633	16.0 (10.2, 22.0)	18,694	16.1 (11.0, 23.0)	< 0.0001
Transplant era							< 0.0001
Before 2001	23,591	37.8	3872	31.8	19,719	38.9	
2001–2005	23,591	17.6	3872	17.7	19,719	17.6	
2005–2010	23,591	21.3	3872	23.2	19,719	20.9	
After 2010	23,591	23.3	3872	27.2	19,719	22.6	
Diabetes, %	23,591	4.5	3872	5.2	19,719	4.3	0.01
CMV, %	23,561	59.3	3869	75.3	19,692	56.2	< 0.0001
HCV, %	19,474	2.7	3343	1.8	16,131	2.7	0.002
Alcohol use, %	23,591	18.0	3872	15.9	19,719	18.4	0.0001
Smoking, %	8773	67.8	1152	60.3	7621	68.9	< 0.0001
Cocaine use, %	2444	44.4	444	59.7	2000	41.0	< 0.0001
Other drug, %	9298	43.5	1544	52.5	7754	41.7	< 0.0001

Data presented as median (Q1, Q3) for continuous variables and N (%) for categorical variables

*EA* European American, *AA* African American, *BMI* Body mass index, *ECD* Extended-criteria donor, *CMV* Cytomegalovirus, *HCV* Hepatitis C virus antibody positive

**Table 2:** Demographic and clinical characteristics of deceased-donor kidney transplant recipients

Variable	All		EA		AA		***P***-value
	N	Median (Q1, Q3), %	N	Median (Q1, Q3), %	N	Median (Q1, Q3), %	
Female, %	47,182	38.10%	23,591	37.10%	23,591	39.00%	< 0.0001
Age, years	47,182	49.0 (39.0, 59.0)	23,591	51 (40.0, 61.0)	23,591	48 (38.0, 57.0)	< 0.0001
BMI, kg/m^2^	40,139	26.8 (23.3, 31.1)	20,211	26.3 (23.0, 30.4)	19,928	27.3 (23.7, 31.6)	< 0.0001
Education							
High school or less, %	31,671	52.5%	16,079	49.7%	15,592	55.4%	< 0.0001
Some college, %	31,671	26.6%	16,079	25.7%	15,592	27.5%	< 0.0001
College graduate, %	31,671	20.8%	16,079	24.5%	15,592	17.0%	< 0.0001
Primary insurance type							
Medicaid, %	39,339	4.1%	19,795	2.9%	19,544	5.4%	< 0.0001
Medicare, %	39,339	65.8%	19,795	60.4%	19,544	71.3%	< 0.0001
Private, %	39,339	28.6%	19,795	35.2%	19,544	21.9%	< 0.0001
Other, %	39,339	1.4%	19,795	1.5%	19,544	1.4%	< 0.0001
Employed, %	41,308	44.4%	20,709	47.6%	20,599	41.2%	< 0.0001
Graft duration, years	47,182	4.1 (1.6, 7.8)	23,591	4.5 (1.8, 8.3)	23,591	3.9 (1.5, 7.2)	< 0.0001
Early failure, %	47,182	7.30%	23,591	6.50%	23,591	8.00%	< 0.0001
Graft failure, %	47,182	48.60%	23,591	46.70%	23,591	50.60%	< 0.0001
Last Peak PRA, %	44,250	4.0 (0.0, 27.0)	22,016	3.0 (0.0, 21.0)	22,234	5.0 (0.0, 32.0)	< 0.0001
Previous transplant, %	46,989	13.2%	23,492	15.2%	23,497	11.2%	< 0.0001
Last Peak PRA > 80%, %	44,250	10.4%	22,016	9.4%	22,234	11.4%	< 0.0001
Previous kidney transplant, %	46,989	11.9%	23,492	13.1%	23,497	10.7%	< 0.0001
Previous dialysis, %	47,182	56.1%	23,591	50.9%	23,591	61.3%	< 0.0001
Time on dialysis, years	21,318	3.7 (2.2, 5.6)	9793	3.1 (1.7, 4.7)	11,525	4.2 (2.7, 6.3)	< 0.0001
Return to dialysis, %	47,182	28.3%	23,591	22.4%	23,591	34.1%	< 0.0001
Death with function, %	47,182	20.4%	23,591	23.8%	23,591	17.1%	< 0.0001
Death, %	47,182	43.6%	23,591	45.0%	23,591	42.1%	< 0.0001
DGF, %	47,125	26.1%	23,568	21.7%	23,557	30.5%	< 0.0001
Discharge serum creatinine, mg/dL	45,784	2.3 (1.5, 4.5)	22,932	2.0 (1.3, 3.7)	22,852	2.6 (1.6, 5.3)	< 0.0001
Cause of kidney failure							
Type 1 diabetes, %	37,099	5.9%	18,717	8.0%	18,382	3.8%	< 0.0001
Type 2 diabetes, %	37,099	15.1%	18,717	13.6%	18,382	16.7%	< 0.0001
Polycystic kidney, %	47,182	6.0%	23,591	9.5%	23,591	2.5%	< 0.0001
Glomerulonephritis, %	47,182	12.9%	23,591	13.7%	23,591	12.1%	< 0.0001
Hypertension, %	47,182	21.4%	23,591	11.7%	23,591	31.0%	< 0.0001
Induction therapy, %	47,182	75.5%	23,591	75.9%	23,591	75.1%	0.05
Acute rejection, %	47,182	1.5%	23,591	1.2%	23,591	1.8%	< 0.0001
Lymphocyte-depleting, %	36,026	4.6%	18,030	4.7%	17,996	4.5%	0.32
Immunosuppression, %	47,141	97.5%	23,577	97.5%	23,564	97.5%	0.74
Immunosuppression class							
Anti-proliferative, %	36,026	87.0%	18,030	86.9%	17,996	87.2%	0.31
Calcineurin Inhibitor, %	36,026	96.6%	18,030	96.6%	17,996	96.6%	0.68
mTOR Inhibitor, %	36,026	7.4%	18,030	7.3%	17,996	7.5%	0.41
Corticosteroid, %	36,026	86.3%	18,030	85.0%	17,996	87.6%	< 0.0001
EPTS	38,657	1.6 (1.0, 2.1)	19,673	1.6 (1.1, 2.1)	18,984	1.5 (1.0, 2.0)	< 0.0001
Other, %	36,026	7.6%	18,030	7.6%	17,996	7.7%	0.79
HCV-positive, %	47,182	5.8%	23,591	4.5%	23,591	7.1%	< 0.0001
Equivalent HLA mismatches (N)	41,940	4.0 (3.0, 5.0)	20,916	4.0 (3.0, 5.0)	21,024	4.0 (3.0, 5.0)	< 0.0001

Data presented as median (Q1, Q3) for continuous variables and N (%) for categorical variables

*EA* European American, *AA* African American, *DGF* Delayed graft failure, *EPTS* Estimated Post Transplant Survival, *HCV* Hepatitis C virus, *HLA* Human leukocyte antigen, *mTOR* Mammalian target of rapamycin, *PRA* Panel reactive antibody

AA and EA DDs had comparable body mass index (BMI) and KDRI. Relative to EA DDs, AA DDs were more likely to be male (64.6% vs. 59.1%), younger (median age 35.0 vs. 40.9 years), cytomegalovirus (CMV) IgG antibody-positive (75.3% vs. 56.2%), and diabetic (5.2% vs. 4.3%). However, AA DDs were less likely to be smokers (60.3% vs. 68.9%) or expanded-criteria donors (12% vs. 14.4%) (Table 1).

Independent of the race/ethnicity of the DD, AA recipients received their transplant at a younger age (median 48.0 vs. 51.0 years), were more likely to have been on dialysis (61.3% vs. 50.9%), and had longer dialysis vintage (4.2 vs. 3.1 years). In addition, AA recipients were less likely to have received a prior transplant (11.2% vs. 15.2%) ordie with a functioning allograft (17.1% vs. 23.8%), but more likely to experience DGF (30.5% vs.21.7%) and had higher rates of acute rejection (1.8% vs. 1.2%) (Table 2). However, rates of immunosuppression medication use and the proportion of KT recipients needing induction therapy were comparable. Supplementary Table 1 show the demographics and clinical characteristics distribution by donor and recipient race.

**Fig. 1:**
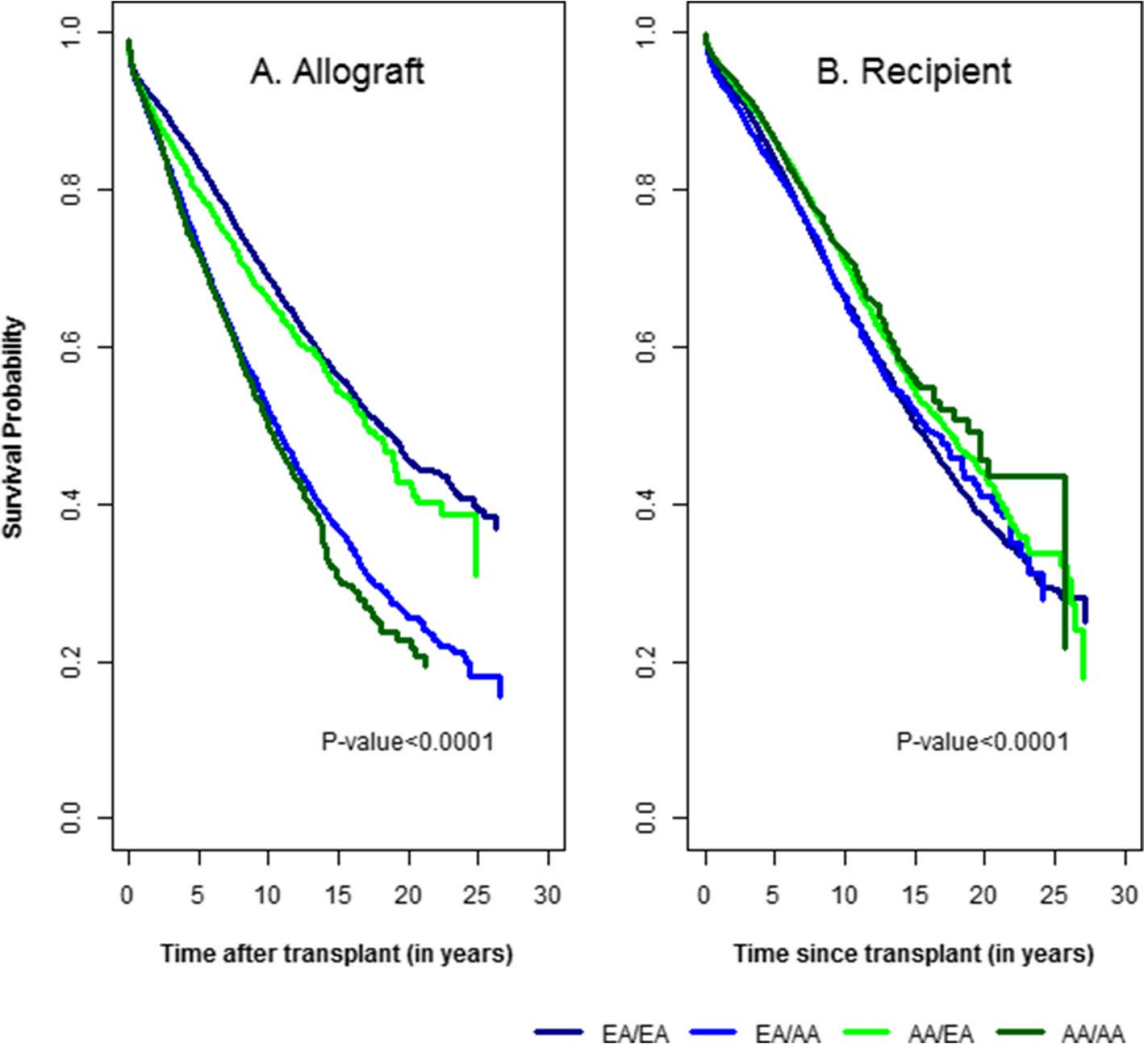
Distribution of allograft survival by type of donor-recipient pair

Fig. 1 displays unadjusted death-censored allograft survival for KT recipients by DRP. Figure 1A shows the unadjusted allograft survival; differences in allograft survival outcomes are apparent between recipients based on race; the top two curves represent DCAS in EA recipients, and the bottom two curves display DCAS in AA recipients. Hazard ratios (HRs) (95% CI) for EA/EA, AA/EA, and EA/AA DRPs, relative to AA/AA pairs, were 0.56 (0.53, 0.60), 0.65 (0.59, 0.70), and 0.96 (0.91, 1.02), respectively. Figure 1B shows unadjusted recipient survival, with mortality treated as a competing risk to allograft failure. At first glance, this graph suggests slightly higher recipient survival rates among AA/AA and EA/AA, compared to AA/EA and EA/EA DRP. However, it is important to keep in mind that AA recipients are approximately 3 years younger than EA recipients. Causes of graft failure did not vary between AA and EA recipients, except for the rate of non-compliance to immunosuppression medication, which was 11.9% among AA recipients, compared to 9.2% for EA recipients.

The five-year DCAS rate improved among all four DRPs during the observation period (Supplementary Table 2). Five-year allograft survival rates in transplants performed after 2010 vs. before 2001 were (0.74 (0.52, 0.90) vs. 0.64 (0.60, 0.67) for AA/AA DRPs, 0.85 (0.76, 0.94) vs. 0.74 (0.71, 0.77) for AA/EA, 0.83 (0.81, 0.86) vs. 0.64 (0.63, 0.65) for EA/AA, and 0.89 (0.87, 0.92) vs. 0.78 (0.77, 0.79) for EA/EA transplantations. Results of the random forest models, which inform the interaction tests that were subsequently performed can be found in Supplementary Table 3.

CPH models showed statistically significant interaction effects between the DRP with the transplant era (0.02), KDRI (*p* = 0.0009), and EPTS (*p* < 0.0001) for DCAS.

The CR analysis helped clarify these results; it showed statistically significant interactions between the DRP and KDRI (*p* < 0.001) for allograft survival, and between the DRP with the KDRI (*p* < 0.0001), EPTS (*p* = 0.009), employment status (*p* < 0.0001) and transplant era (*p* < 0.0001) with kidney recipient mortality. Table 3 shows HRs for overall DCAS according to employment status and assuming no change in KDRI and EPTS. With employment EA/EA DRPs saw consistent improvement over time; for transplantations performed after 2010, HRs ranged from 0.42 (0.37, 0.47) to 0.46 (0.41, 0.51) for employed recipients and from 0.52 (0.48, 0.58) to 0.57 (0.52, 063) for unemployed recipients. Similar improvements were also observed with AA/EA pairs. However, for EA/AA DRPs, significant improvement in the overall DCAS was observed only post-2010 DDKTs, and the overall improvement was significantly smaller; HRs were 0.78 (0.66, 0.92) for EA/AA DRPs, compared to 0.42 (0.38, 0.47) for EA/EA’s.

**Table 3:** Hazard ratio and 95% confidence interval (HR (95% CI)) for death-censored kidney allograft failure by DRP and transplant era, depending on employment status, and change in KDRI and EPTS score

DRP	Transplant era	Employed				Unemployed			
		KDRI = 0, EPTS = 0	KDRI = 0, EPTS = 0.25	KDRI = 0.25, EPTS = 0	KDRI = 0.25, EPTS = 0.25	KDRI = 0, EPTS = 0	KDRI = 0, EPTS = 0.25	KDRI = 0.25, EPTS = 0	KDRI = 0.25, EPTS = 0.25
AA/AA	2001–2005	1.26 (0.97, 1.63)	1.36 (1.06, 1.73)	1.17 (0.91, 1.50)	1.26 (1.00, 1.60)	1.43 (1.10, 1.85)	1.54 (1.20, 1.97)	1.46 (1.14, 1.87)	1.57 (1.25, 1.98)
AA/AA	2005–2010	1.08 (0.82, 1.41)	1.16 (0.91, 1.50)	1.00 (0.77, 1.30)	1.08 (0.85, 1.38)	1.22 (0.94, 1.60)	1.32 (1.03, 1.70)	1.25 (0.97, 1.62)	1.35 (1.06, 1.72)
AA/AA	After 2010	0.93 (0.70, 1.25)	1.01 (0.77, 1.32)	0.87 (0.66, 1.15)	0.94 (0.72, 1.22)	1.06 (0.80, 1.41)	1.26 (0.96, 1.65)	1.08 (0.82, 1.43)	1.17 (0.90, 1.52)
AA/AA	Before 2001	Reference							
AA/EA	2001–2005	0.65 (0.49, 0.85)	0.72 (0.56, 0.93)	0.64 (0.49, 0.83)	0.71 (0.56, 0.91)	0.78 (0.59, 1.03)	0.87 (0.67, 1.13)	0.80 (0.62, 1.03)	0.89 (0.70, 1.13)
AA/EA	2005–2010	0.58 (0.44, 0.76)	0.65 (0.50, 0.84)	0.57 (0.44, 0.75)	0.64 (0.50, 0.82)	0.70 (0.53, 0.93)	0.78 (0.60, 1.02)	0.71 (0.55, 0.93)	0.80 (0.62, 1.02)
AA/EA	After 2010	0.49 (0.36, 0.66)	0.54 (0.41, 0.72)	0.48 (0.36, 0.64)	0.54 (0.41, 0.70)	0.59 (0.43, 0.80)	0.68 (0.51, 0.90)	0.60 (0.45, 0.80)	0.67 (0.51, 0.87)
AA/EA	Before 2001	Reference							
EA/AA	2001–2005	1.29 (1.11, 1.49)	1.36 (1.18, 1.57)	1.21 (1.05, 1.40)	1.28 (1.11, 1.47)	1.36 (1.17, 1.58)	1.44 (1.24, 1.66)	1.28 (1.10, 1.48)	1.35 (1.17, 1.55)
EA/AA	2005–2010	1.03 (0.89, 1.20)	1.09 (0.94, 1.26)	0.97 (0.84, 1.12)	1.02 (0.89, 1.18)	1.09 (0.93, 1.27)	1.15 (0.99, 1.33)	1.02 (0.88, 1.19)	1.08 (0.93, 1.25)
EA/AA	After 2010	0.78 (0.66, 0.92)	0.82 (0.70, 0.96)	0.73 (0.62, 0.86)	0.77 (0.66, 0.90)	0.82 (0.69, 0.97)	0.87 (0.74, 1.02)	0.77 (0.65, 0.91)	0.81 (0.69, 0.95)
EA/AA	Before 2001	Reference							
EA/EA	2001–2005	0.57 (0.52, 0.62)	0.62 (0.57, 0.68)	0.57 (0.52, 0.62)	0.62 (0.57, 0.67)	0.71 (0.67, 0.76)	0.78 (0.73, 0.83)	0.71 (0.67, 0.75)	0.77 (0.72, 0.82)
EA/EA	2005–2010	0.52 (0.48, 0.57)	0.57 (0.52, 0.62)	0.52 (0.48, 0.57)	0.57 (0.52, 0.62)	0.65 (0.61, 0.70)	0.71 (0.66, 0.76)	0.65 (0.61, 0.69)	0.71 (0.66, 0.75)
EA/EA	After 2010	0.42 (0.38, 0.47)	0.46 (0.41, 0.51)	0.42 (0.38, 0.47)	0.46 (0.41, 0.51)	0.53 (0.48, 0.58)	0.57 (0.52, 0.63)	0.52 (0.48, 0.58)	0.57 (0.52, 0.63)
EA/EA	Before 2001	Reference							

Models were adjusted for recipient age at transplant, recipient sex, presence of DGF, previous dialysis, education level, recipient equivalent HLA mismatch, peak PRA, recipient HCV status, cold ischemia time, donor age, donor CMV status, use of immunosuppressants, including use of lymphocyte depleting drugs, mTOR inhibitors and steroids

*AA* African American, *EA* European American

**Fig. 2:**
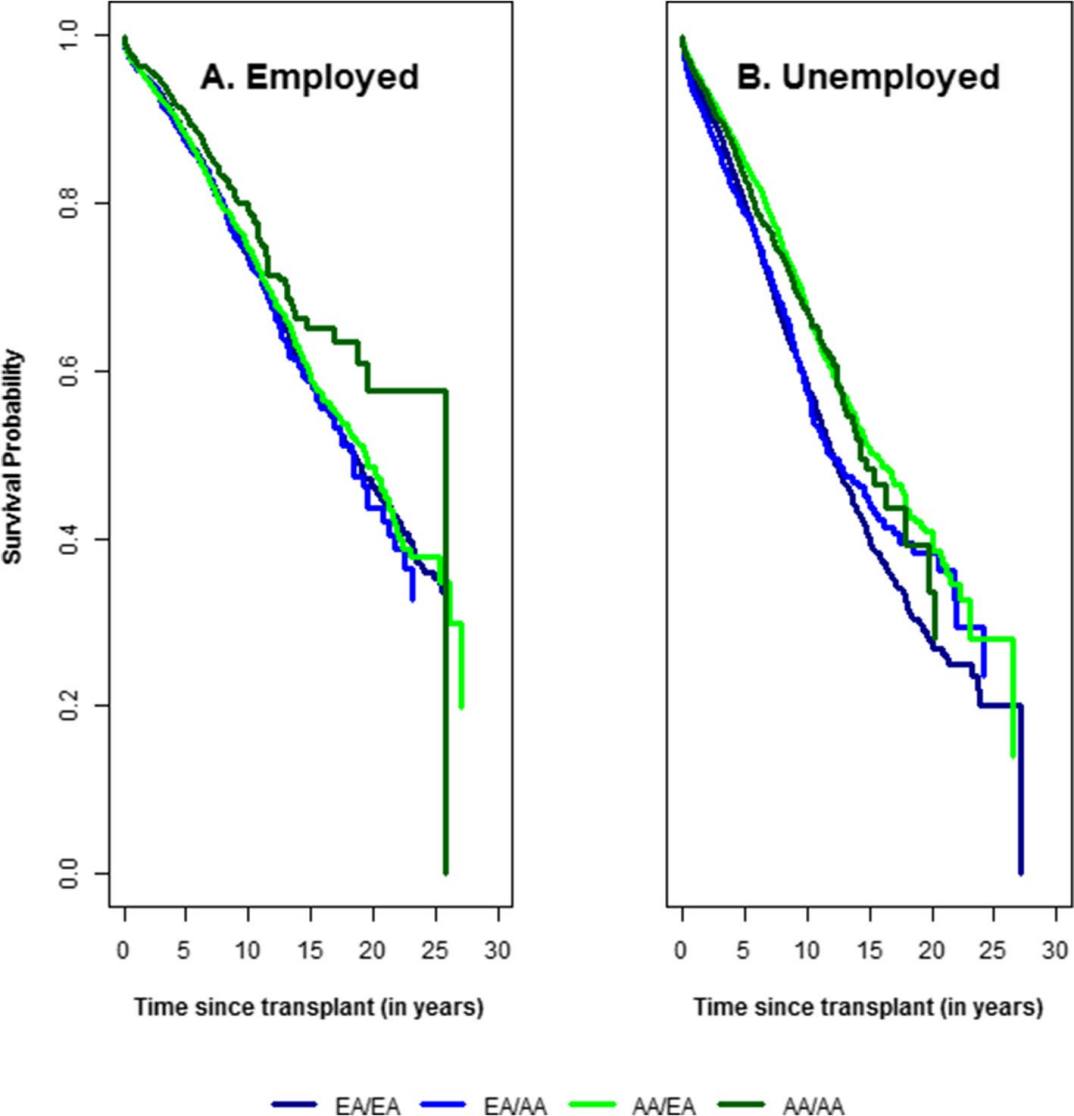
Effect of employment on mortality by type of donor-recipient pair

Table 4 shows HRs for the effect of DRP, KDRI, EPTS, and transplant era and employment status on recipient mortality with allograft failure as a CR. For transplantations performed before 2001 and assuming no change in KDRI and EPTS over time, reductions in mortality were observed among all four DRPs for employed DDKT. HRs for the post 2010 transplant era were 0.24 (0.13, 0.43), 0.27 (0.17, 0.45), 0.20 (0.14, 0.28), 0.24 (0.19, 0.32) for AA/AA, AA/EA, EA/AA and AA/AA DRPs, respectively. In contrast, HRs for mortality were higher among unemployed recipients; 0.50 (0.29, 0.87), 0.55 (0.35, 0.87), 0.32 (0.24, 0.42), and 0.49 (0.43, 0.57) among these 4 DRPs, assuming no change in KDRI and EPTS. Figure 2 shows the disparity in recipient mortality according to employment status and DRP.

**Table 4:** Hazard ratio and 95% confidence interval (HR (95% CI)) for mortality as a competing risk to allograft failure by DRP and transplant era, depending on employment status, and change in KDRI and EPTS score

DRP	Transplant era	Employed				Unemployed			
		KDRI = 0, EPTS = 0	KDRI = 0, EPTS = 0.25	KDRI = 0.25, EPTS = 0	KDRI = 0.25, EPTS = 0.25	KDRI = 0, EPTS = 0	KDRI = 0, EPTS = 0.25	KDRI = 0.25, EPTS = 0	KDRI = 0.25, EPTS = 0.25
AA/AA	2001–2005	0.33 (0.19, 0.55)	0.36 (0.22, 0.60)	0.38 (0.24, 0.63)	0.43 (0.27, 0.68)	0.63 (0.39, 1.02)	0.70 (0.44, 1.11)	0.70 (0.44, 1.11)	0.78 (0.50, 1.20)
AA/AA	2005–2010	0.23 (0.13, 0.39)	0.25 (0.15, 0.42)	0.27 (0.16, 0.44)	0.30 (0.18, 0.48)	0.51 (0.31, 0.85)	0.57 (0.35, 0.92)	0.57 (0.36, 0.92)	0.64 (0.41, 1)
AA/AA	After 2010	0.24 (0.13, 0.43)	0.27 (0.15, 0.47)	0.28 (0.16, 0.5)	0.31 (0.18, 0.54)	0.50 (0.29, 0.87)	0.56 (0.33, 0.95)	0.56 (0.33, 0.95)	0.63 (0.38, 1.03)
AA/AA	Before 2001	Reference							
AA/EA	2001–2005	0.38 (0.25, 0.60)	0.43 (0.28, 0.66)	0.45 (0.30, 0.69)	0.51 (0.34, 0.76)	0.71 (0.47, 1.06)	0.79 (0.53, 1.17)	0.80 (0.55, 1.18)	0.90 (0.62, 1.30)
AA/EA	2005–2010	0.28 (0.18, 0.45)	0.32 (0.20, 0.50)	0.34 (0.22, 0.52)	0.38 (0.25, 0.58)	0.62 (0.41, 0.94)	0.69 (0.46, 1.03)	0.70 (0.47, 1.04)	0.79 (0.54, 1.15)
AA/EA	After 2010	0.27 (0.17, 0.45)	0.31 (0.19, 0.50)	0.32 (0.20, 0.52)	0.36 (0.23, 0.58)	0.55 (0.35, 0.87)	0.61 (0.39, 0.96)	0.62 (0.40, 0.96)	0.70 (0.46, 1.06)
AA/EA	Before 2001	Reference							
EA/AA	2001–2005	0.33 (0.25, 0.45)	0.35 (0.26, 0.47)	0.41 (0.31, 0.55)	0.43 (0.33, 0.57)	0.49 (0.38, 0.63)	0.52 (0.40, 0.66)	0.58 (0.45, 0.74)	0.61 (0.48, 0.77)
EA/AA	2005–2010	0.22 (0.16, 0.31)	0.24 (0.17, 0.32)	0.28 (0.2, 0.37)	0.29 (0.22, 0.39)	0.39 (0.29, 0.51)	0.41 (0.31, 0.53)	0.45 (0.35, 0.59)	0.48 (0.37, 0.62)
EA/AA	After 2010	0.20 (0.14, 0.28)	0.21 (0.15, 0.3)	0.24 (0.17, 0.34)	0.26 (0.18, 0.36)	0.32 (0.24, 0.42)	0.33 (0.25, 0.44)	0.37 (0.28, 0.49)	0.39 (0.3, 0.52)
EA/AA	Before 2001	Reference							
EA/EA	2001–2005	0.36 (0.29, 0.44)	0.40 (0.32, 0.49)	0.43 (0.35, 0.52)	0.47 (0.39, 0.57)	0.67 (0.61, 0.74)	0.74 (0.66, 0.82)	0.76 (0.69, 0.83)	0.84 (0.76, 0.92)
EA/EA	2005–2010	0.29 (0.23, 0.36)	0.32 (0.26, 0.40)	0.35 (0.28, 0.43)	0.38 (0.31, 0.47)	0.63 (0.57, 0.71)	0.70 (0.63, 0.78)	0.72 (0.65, 0.80)	0.79 (0.72, 0.88)
EA/EA	After 2010	0.24 (0.19, 0.32)	0.27 (0.21, 0.35)	0.29 (0.23, 0.38)	0.32 (0.25, 0.41)	0.49 (0.43, 0.57)	0.54 (0.47, 0.63)	0.56 (0.49, 0.65)	0.62 (0.53, 0.71)
EA/EA	Before 2001	Reference							

Models were adjusted for recipient age at transplant, recipient sex, presence of DGF, previous dialysis, education level, recipient equivalent HLA mismatch, peak PRA, recipient HCV status, cold ischemia time, donor age, donor CMV status, use of immunosuppressants, including use of lymphocyte depleting drugs, mTOR inhibitors and steroids

*AA* African American, *EA* European American

## Discussion

Donor characteristics contribute to racial disparities in outcomes following DDKT [2, 23, 37]. The present study evaluated recipient factors potentially affecting ethnic disparities in DDKT outcomes using a unique donor-matched design that controlled for genetic differences in transplanted kidneys, which allowed us to limit the impact of donor characteristics on DDKT outcomes, including many donor factors not available in the OPTN registry.

The analysis included 47,182 total kidney transplantations, 3872 involving AA DDs. As such, it is the most extensive analysis of its kind. Transplants resulting from the four possible DRPs had different DCAS, with EA recipients having better overall allograft survival than AA, independent from DD race/ethnicity. Analyses suggest that multiple factors contribute to kidney allograft outcomes. Some of the reported associations were described previously, including the well-known effects of DGF, serum creatinine at hospital discharge, recipient age, KDRI, EPTS, immunosuppressant medication, transplant era, donor age, etc. [7, 38, 39] However, these effects are not modified by the DRP.

Employment status, KDRI, and EPTS interacted with DRP to affect DDKT outcomes. Unemployed recipients had worse DDKT allograft survival and mortality. Employment status was obtained before kidney transplantation. Recipients who reported working a full-time or a part-time job was considered employed; all others were considered unemployed, independently of the reason for not working. The HR estimates among unemployed recipients were almost twice those observed among employed recipients for mortality, although there was a minor overlap between confidence intervals in some cases.

Employment status at transplantation was the only socioeconomic variable that showed significant interaction effects with the DRP. The absence of independent effects of educational attainment and insurance status probably reflects the careful screening process of potential recipients by transplant programs. In contrast, employment status is rarely invoked as a reason to preclude active status of wait-listed transplant candidates in the US, despite its potential adverse effect on the ability to afford medications or access health insurance, especially after expiration of the 36-month post-transplant coverage provided by the Center for Medicare and Medicaid Services. The newly passed Immuno Bill indefinitely extends Medicare coverage of immunosuppressive drugs for KT recipients and may help reduce disparities in long-term allograft survival. However, employment status may be a broader measure of social determinants of health with a clear association between unemployment, job loss, and retirement with poor outcomes.

In contrast, employment contributes to better physical health [40–42]. Unemployed individuals, independent of race/ethnicity, more often report feelings of depression and anxiety and high blood pressure, and tend to have higher rates of stroke, heart attack, and heart disease [43–45]. Unlike the composite scores considered in these analyses, employment status is a modifiable factor. Specific steps can be taken to understand how it affects outcomes among DDKT recipients and mitigate its effects.

Some measures reported in these analyses (e.g., KDRI and EPTS) are relatively new and were not previously part of the kidney allocation process. However, their utilization in these analyses ensures that comparisons across transplant eras are appropriate. KDRI includes donor race and other donor demographic and clinical characteristics. EPTS depends on recipient age, diabetes status, prior organ transplantations, and previous time on dialysis. Including these scores, the DRP, and the other variables in these models may have induced some collinearity. However, the random forests models are robust to multicollinearity. The KDRI score for AA donors is multiplied by a factor of 1.2, regardless of donor age, sex, and presence of other comorbidities. However, AA deceased donors were more likely to be younger and males such that the distributions of KDRI scores were comparable between AA and EA donors. The inclusion of these variables in the models was meant to help determine how socioeconomic and social determinants of health factors, which may interact with these scores, affect kidney transplant outcomes among AA and EA recipients.

Limitations of this report include potential underreporting in the SRTR database of various outcomes (e.g., DGF), mischaracterization of race and ethnicity, and viral infections, whose effects on KT outcomes were not initially recognized [46]. Analyses used registry data that were not collected for research purposes; therefore, some variables (e.g., employment status, medication use) may be incomplete and might not have been rigorously collected. However, it is unclear when the ongoing prospective APOL1 Long-term Kidney Transplantation Outcomes (APOLLO) study will accumulate enough events to address these questions [47]. These analyses provide some preliminary results that can be explored in other datasets.

Also, the study compared DDKT outcomes over more than 30 years, such that the standard of care and ways that measurements were collected and reported to the SRTR may have changed over time. However, focusing on four transplant eras should reduce these effects and their likelihood for confounding. These analyses were performed in a non-random subset of the SRTR data that may not have provided a representative sample of the distribution of outcomes observed among all DDKT recipients. For multiple reasons, including a greater need for kidney transplants in AA, lower rate of living kidney donation among AA, higher rates of HLA matching among individuals with recent African ancestry, waitlisted AA are more likely to receive AA DDKTs. Therefore, AA/AA DRP represents a significant proportion of all DDKTs [7, 48, 49].

## Conclusion

AA recipients of kidney transplants from AA DDs had significantly shorter kidney allograft survival than EA recipients of AA DD kidneys and AA recipients of EA DD kidneys. Mortality among DDKT recipients remains high, especially among unemployed recipients, and does not appear to have changed since the early 2000s among unemployed AA recipients. Unemployment is associated with poorer outcomes among DDKT recipients, independent of race/ethnicity; however, its effects appeared to be consistently worse for AA DDKT recipients. Thus, improving outcomes for transplant recipients will require an improved understanding of the mechanisms by which socioeconomic factors, such as unemployment, adversely affect outcomes in the United States.

## Supplementary Information

**Additional file 1: Supplementary Table 1.** Demographic and clinical characteristics by race/ethnicity of the donor-recipient pair. **Supplementary Table 2.** Five‐year death‐censored kidney allograft survival probability and 95% confidence interval by DRP and transplant era. **Supplementary Table 3.** Predictor ranking based on variable importance for death-censored kidney allograft survival and allograft survival with mortality as a competing risk.

## Abbreviations

AA: African American
APOL1: Apolipoprotein L1
APOLLO: APOL1 Long-term Kidney Transplantation Outcomes
BMI: Body mass index
BP: Blood pressure
CIT: cold ischemia time
CIT: Confidence interval
CKD: Chronic kidney disease
CMV: Cytomegalovirus
CPH: Cox proportional hazard model
CR: Competing risk
CVD: Cardiovascular disease
DCAS: Death-censored allograft survival
DD: Deceased donors
DDKT: Deceased donor kidney transplantation
DGF: Delayed graft function
DNA: Deoxyribonucleic acid
DRP: Donor/recipient pairs
EA: European American
EPTS: Estimated post-transplant survival
ESKD: End-stage kidney disease
HLA: Human Leucocyte antigen
HR: Hazard ratio
HRSA: Health Resources and Services Administration
KDRI: Kidney Donor Risk Index
KT: Kidney transplant / kidney transplantation
NUDT7: Nudix hydrolase 7 gene
OPTN: Organ Procurement and Transplantation Network
PRA: Panel reactive antibodies
RSF: Random survival forest
SEC63: Translocation protein SEC63 homolog
SNP: Single nucleotide polymorphism
SRTR: Scientific Registry for Transplant Outcomes
T2D: Type 2 diabetes
UMOD: Uromodulin
UNOS: United Network for Organ Sharing
USRDS: United States Renal Data System
VIMP: Variable importance
