# Supplementary material for: Employment status at transplant influences ethnic disparities in outcomes after deceased donor kidney transplantation
Source: BMC Nephrol. 2022 Jan 3;23:6. doi: 10.1186/s12882-021-02631-4 (PMC8722061; doi:10.1186/s12882-021-02631-4)
Supplement: Supplementary file 1 — Additional file 1: Supplementary Table 1. Demographic and clinical characteristics by race/ethnicity of the donor-recipient pair. Supplementary Table 2. Five‐year death‐censored kidney allograft survival probability and 95% confidence interval by DRP and transplant era. Supplementary Table 3. Predictor ranking based on variable importance for death-censored kidney allograft survival and allograft survival with mortality as a competing risk. [file 12882_2021_2631_MOESM1_ESM.docx]

**Supplementary Table 1: Demographic and clinical characteristics by race/ethnicity of the donor-recipient pair**

| **Variable** | **All** | | **AA donors** | | | | | **EA donors** | | | | | | **Overall  P-value** |
| --- | --- | --- | --- | --- | --- | --- | --- | --- | --- | --- | --- | --- | --- | --- |
|  |  |  | **AA recipients** | | **EA recipients** | |  | **AA recipients** | | **EA recipients** | | |  |  |
|  | **N** | **Median (Q1, Q3), %** | **N** | **Median (Q1, Q3), %** | **N** | **Median (Q1, Q3), %** | **P-value** | **N** | **Median (Q1, Q3), %** | **N** | **Median (Q1, Q3), %** | | **P-value** |  |
| Female, % | 47182 | 38.1% | 3872 | 40.1% | 3872 | 38.3% | 0.1 | 19719 | 38.8% | 19719 | 36.9% | | <0.0001 | <0.0001 |
| Age, years | 47182 | 49.0 (39.0,59.0) | 3872 | 48.0 (38.0, 58.0) | 3872 | 51.0 (40.0, 60.0) | <0.0001 | 19719 | 48.0 (38.0, 57.0) | 19719 | 51.0 (40.0, 61.0) | | <0.0001 | <0.0001 |
| BMI, kg/m^2^ | 40139 | 26.8 (23.3,31.1) | 3297 | 27.1 (23.7, 31.3) | 3374 | 26.1 (22.9, 30.2) | <0.0001 | 16631 | 27.3 (23.7, 31.6) | 16837 | 26.4 (23.1, 30.5) | | <0.0001 | <0.0001 |
| Education |  | | | | | | | | | | | | | |
| High school or less, % | 31671 | 52.5% | 2720 | 54.5% | 2803 | 48.1% | <0.0001 | 12872 | 55.6% | 13276 | 50.1% | | <0.0001 | <0.0001 |
| Some college, % | 31671 | 26.6% | 2720 | 28.2% | 2803 | 24.8% | <0.0001 | 12872 | 27.4% | 13276 | 25.9% | | <0.0001 | <0.0001 |
| College graduate, % | 31671 | 20.8% | 2720 | 17.3% | 2803 | 27.1% | <0.0001 | 12872 | 17.0% | 13276 | 24.0% | | <0.0001 | <0.0001 |
| Primary insurance type |  | | | | | | | | | | | | | |
| Medicaid, % | 39339 | 4.1% | 3360 | 4.8% | 3406 | 3.0% | <0.0001 | 16184 | 5.5% | 16389 | 2.9% | | <0.0001 | <0.0001 |
| Medicare, % | 39339 | 65.8% | 3360 | 73.9% | 3406 | 58.7% | <0.0001 | 16184 | 70.8% | 16389 | 60.8% | | <0.0001 | <0.0001 |
| Private, % | 39339 | 28.6% | 3360 | 20.4% | 3406 | 36.9% | <0.0001 | 16184 | 22.2% | 16389 | 34.9% | | <0.0001 | <0.0001 |
| Other, % | 39339 | 1.4% | 3360 | 1.0% | 3406 | 1.4% | <0.0001 | 16184 | 1.5% | 16389 | 1.5% | | <0.0001 | <0.0001 |
| Employed, % | 41308 | 44.4% | 3359 | 38.4% | 3339 | 46.5% | <0.0001 | 17240 | 41.8% | 17370 | 47.8% | | <0.0001 | <0.0001 |
| Graft duration, years | 47182 | 4.1 (1.6,7.8) | 3872 | 3.7 (1.6, 6.9) | 3872 | 3.9 (1.4, 7.8) | 0.004 | 19719 | 4.0 (1.5, 7.3) | 19719 | 4.7 (1.8, 8.5) | | <0.0001 | <0.0001 |
| Early failure, % | 47182 | 7.3% | 3872 | 7.9% | 3872 | 7.3% | 0.35 | 19719 | 8.0% | 19719 | 6.4% | | <0.0001 | <0.0001 |
| Graft failure, % | 47182 | 48.6% | 3872 | 48.6% | 3872 | 46.0% | 0.03 | 19719 | 51.0% | 19719 | 46.8% | | <0.0001 | <0.0001 |
| Last Peak PRA, % | 44250 | 4.0 (0.0,27.0) | 3656 | 7.0 (0.0, 44.0) | 3596 | 3.0 (0.0, 22.0) | <0.0001 | 18578 | 5.0 (0.0, 30.0) | 18420 | 3.0 (0.0, 21.0) | | <0.0001 | <0.0001 |
| Previous transplant, % | 46989 | 13.2% | 3867 | 12.2% | 3860 | 14.0% | 0.02 | 19630 | 11.0% | 19632 | 15.5% | | <0.0001 | <0.0001 |
| Last Peak PRA >80%, % | 44250 | 10.4% | 3656 | 14.3% | 3596 | 9.4% | <0.0001 | 18578 | 10.9% | 18420 | 9.4% | | <0.0001 | <0.0001 |
| Previous kidney transplant, % | 46989 | 11.9% | 3867 | 11.7% | 3860 | 11.2% | 0.49 | 19630 | 10.5% | 19632 | 13.5% | | <0.0001 | <0.0001 |
| Previous dialysis, % | 47182 | 56.1% | 3872 | 63.7% | 3872 | 51.3% | <0.0001 | 19719 | 60.8% | 19719 | 50.8% | | <0.0001 | <0.0001 |
| Time on dialysis, years | 21318 | 3.7 (2.2,5.6) | 2055 | 4.4 (2.7, 6.5) | 1688 | 3.1 (1.7, 4.7) | <0.0001 | 9470 | 4.2 (2.6, 6.2) | 8105 | 3.1 (1.7, 4.7) | | <0.0001 | <0.0001 |
| Return to dialysis, % | 47182 | 28.3% | 3872 | 33.2% | 3872 | 21.8% | <0.0001 | 19719 | 34.3% | 19719 | 22.5% | | <0.0001 | <0.0001 |
| Death with function, % | 47182 | 20.4% | 3872 | 15.6% | 3872 | 22.3% | <0.0001 | 19719 | 17.4% | 19719 | 24.1% | | <0.0001 | <0.0001 |
| Death, % | 47182 | 43.6% | 3872 | 39.1% | 3872 | 42.4% | 0.003 | 19719 | 42.7% | 19719 | 45.5% | | <0.0001 | <0.0001 |
| DGF, % | 47125 | 26.1% | 3868 | 26.3% | 3868 | 20.1% | <0.0001 | 19689 | 31.4% | 19700 | 22.0% | | <0.0001 | <0.0001 |
| Discharge serum creatinine, mg/dL | 45784 | 2.3 (1.5,4.5) | 3763 | 2.6 (1.6, 4.9) | 3751 | 1.9 (1.3, 3.5) | <0.0001 | 19089 | 2.6 (1.6, 5.4) | 19181 | 2.0 (1.4, 3.7) | | <0.0001 | <0.0001 |
| Cause of kidney failure |  | | | | | | | | | | | | | |
| Type 1 diabetes, % | 37099 | 5.9% | 3173 | 4.7% | 3228 | 12.4% | <0.0001 | 15209 | 3.6% | 15489 | 7.1% | | <0.0001 | <0.0001 |
| Type 2 diabetes, % | 37099 | 15.1% | 3173 | 17.2% | 3228 | 14.2% | 0.0008 | 15209 | 16.6% | 15489 | 13.4% | | <0.0001 | <0.0001 |
| Polycystic kidney, % | 47182 | 6.0% | 3872 | 2.6% | 3872 | 8.7% | <0.0001 | 19719 | 2.5% | 19719 | 9.7% | | <0.0001 | <0.0001 |
| Glomerulonephritis, % | 47182 | 12.9% | 3872 | 12.6% | 3872 | 12.4% | 0.86 | 19719 | 12.1% | 19719 | 13.9% | | <0.0001 | <0.0001 |
| Hypertension, % | 47182 | 21.4% | 3872 | 33.7% | 3872 | 12.3% | <0.0001 | 19719 | 30.5% | 19719 | 11.6% | | <0.0001 | <0.0001 |
| Induction therapy, % | 47182 | 75.5% | 3872 | 77.1% | 3872 | 77.2% | 0.94 | 19719 | 74.7% | 19719 | 75.6% | | 0.03 | 0.0003 |
| Acute rejection, % | 47182 | 1.5% | 3872 | 1.6% | 3872 | 1.2% | 0.17 | 19719 | 1.9% | 19719 | 1.2% | | <0.0001 | <0.0001 |
| Lymphocyte-depleting, % | 36026 | 4.6% | 2924 | 4.8% | 2870 | 5.5% | 0.22 | 15072 | 4.4% | 15160 | 4.5% | | 0.59 | 0.05 |
| Immunosuppression, % | 47141 | 97.5% | 3868 | 97.7% | 3867 | 96.8% | 0.03 | 19696 | 97.4% | 19710 | 97.6% | | 0.16 | 0.03 |
| Immunosuppression class |  | | | | | | | | | | | | | |
| Anti-proliferative, % | 36026 | 87.0% | 2924 | 85.9% | 2870 | 86.4% | 0.61 | 15072 | 87.5% | 15160 | 86.9% | 0.18 | | 0.08 |
| Calcineurin Inhibitor, % | 36026 | 96.6% | 2924 | 96.4% | 2870 | 96.7% | 0.56 | 15072 | 96.6% | 15160 | 96.6% | 0.85 | | 0.94 |
| mTOR Inhibitor, % | 36026 | 7.4% | 2924 | 7.8% | 2870 | 8.0% | 0.72 | 15072 | 7.5% | 15160 | 7.1% | 0.29 | | 0.26 |
| Corticosteroid, % | 36026 | 86.3% | 2924 | 85.80% | 2870 | 84.0% | 0.05 | 15072 | 88.0% | 15160 | 85.3% | <0.0001 | | <0.0001 |
| EPTS | 38657 | 1.6 (1.0,2.1) | 3157 | 1.5 (1.0, 2.0) | 3269 | 1.6 (1.1, 2.1) | 0.0005 | 15827 | 1.5 (1.0, 2.0) | 16404 | 1.6 (1.1, 2.1) | <0.0001 | | <0.0001 |
| Other, % | 36026 | 7.6% | 2924 | 8.7% | 2870 | 8.4% | 0.66 | 15072 | 7.5% | 15160 | 7.4% | 0.94 | | 0.04 |
| HCV-positive, % | 47182 | 5.8% | 3872 | 7.1% | 3872 | 4.7% | <0.0001 | 19719 | 7.1% | 19719 | 4.5% | <0.0001 | | <0.0001 |
| Equivalent HLA mismatches (N) | 41940 | 4.0 (3.0,5.0) | 3568 | 4.0 (3.0, 5.0) | 3523 | 5.0 (4.0, 5.0) | <0.0001 | 17456 | 4.0 (3.0, 5.0) | 17393 | 4.0 (3.0, 5.0) | <0.0001 | | <0.0001 |

**Supplementary Table 2: Five‐year death‐censored kidney allograft survival probability and 95% confidence interval by DRP and transplant era**

| **DRP** | **Transplant era** | | | |
| --- | --- | --- | --- | --- |
|  | **Before 2001** | **2001-2005** | **2006-2010** | **Post-2010** |
| **AA/AA** | 0.64 (0.61, 0.67) | 0.73 (0.70, 0.77) | 0.76 (0.73, 0.79) | 0.74 (0.61, 0.9) |
| **AA/EA** | 0.74 (0.71, 0.77) | 0.8 (0.77, 0.83) | 0.81 (0.78, 0.84) | 0.85 (0.76, 0.94) |
| **EA/AA** | 0.64 (0.63, 0.65) | 0.74 (0.73, 0.76) | 0.8 (0.78, 0.81) | 0.83 (0.81, 0.86) |
| **EA/EA** | 0.78 (0.77, 0.79) | 0.86 (0.84, 0.87) | 0.87 (0.86, 0.89) | 0.89 (0.87, 0.92) |

DRP: donor‐recipient pair; AA: African American; EA: European American

**Supplementary Table 3: Predictor ranking based on variable importance for death-censored kidney allograft survival and allograft survival with mortality as a competing risk**

| **Variables** | **Death-censored allograft failure** | | | **Competing risk model** | | | | | | |
| --- | --- | --- | --- | --- | --- | --- | --- | --- | --- | --- |
|  |  |  |  | **All** | **Allograft failure** | | | **Death with allograft function** | | |
|  | **All** | **Discovery** | **Validation** |  | **All** | **Discovery** | **Validation** | **All** | **Discovery** | **Validation** |
| Transplant era (Before 2001) | ***1*** | 1 | 1 | ***1*** | ***1*** | 1 | 1 | ***2*** | 2 | 2 |
| Recipient discharge serum creatinine | ***2*** | 2 | 2 | ***7*** | ***3*** | 3 | 4 | ***47*** | 39 | 52 |
| Recipient age at transplant | ***3*** | 3 | 4 | ***2*** | ***5*** | 6 | 5 | ***1*** | 1 | 1 |
| Delayed graft function | ***4*** | 4 | 3 | ***4*** | ***2*** | 2 | 2 | ***12*** | 14 | 17 |
| Donor-recipient pair | ***5*** | 6 | 5 | ***13*** | ***11*** | 10 | 10 | ***20*** | 11 | 36 |
| Use of immunosuppression drug | ***6*** | 7 | 6 | ***9*** | ***10*** | 11 | 8 | ***7*** | 7 | 6 |
| Donor age at death | ***7*** | 5 | 7 | ***8*** | ***6*** | 5 | 7 | ***10*** | 10 | 9 |
| KDRI | ***8*** | 8 | 9 | ***5*** | ***4*** | 4 | 3 | ***6*** | 5 | 7 |
| EPTS | ***9*** | 9 | 8 | ***3*** | ***8*** | 8 | 9 | ***3*** | 3 | 3 |
| Use of lymphocyte-depleting drug | ***10*** | 10 | 10 | ***26*** | ***17*** | 20 | 15 | ***40*** | 36 | 50 |
| Previous dialysis | ***11*** | 13 | 14 | ***12*** | ***13*** | 15 | 14 | ***11*** | 12 | 8 |
| Transplant era (2001-2005) | ***12*** | 19 | 18 | ***6*** | ***7*** | 9 | 6 | ***4*** | 4 | 5 |
| Immunosuppression class (Other) | ***13*** | 12 | 11 | ***16*** | ***14*** | 18 | 12 | ***16*** | 20 | 22 |
| Extended-criteria donor | ***14*** | 11 | 16 | ***17*** | ***16*** | 14 | 20 | ***25*** | 23 | 53 |
| Diabetes mellitus status | ***15*** | 16 | 12 | ***11*** | ***12*** | 12 | 13 | ***5*** | 8 | 4 |
| Transplant era (2006-2010) | ***16*** | 18 | 15 | ***10*** | ***9*** | 7 | 11 | ***8*** | 6 | 11 |
| Number of HLA mismatches | ***17*** | 14 | 13 | ***19*** | ***18*** | 24 | 21 | ***24*** | 31 | 18 |
| Acute rejection | ***18*** | 17 | 30 | ***43*** | ***52*** | 38 | 35 | ***44*** | 41 | 38 |
| Candidate last peak PRA | ***19*** | 15 | 23 | ***31*** | ***26*** | 31 | 19 | ***49*** | 40 | 49 |
| Immunosuppression class (mTOR) | ***20*** | 20 | 24 | ***20*** | ***20*** | 26 | 23 | ***23*** | 42 | 14 |
| Polycystic kidney | ***21*** | 25 | 22 | ***15*** | ***15*** | 13 | 22 | ***14*** | 13 | 16 |
| Donor hypertension | ***22*** | 21 | 32 | ***21*** | ***22*** | 19 | 16 | ***22*** | 47 | 13 |
| Number of HLA mismatches equivalent | ***23*** | 22 | 19 | ***25*** | ***23*** | 23 | 24 | ***29*** | 16 | 54 |
| Recipient drug induction | ***24*** | 24 | 17 | ***24*** | ***21*** | 21 | 17 | ***28*** | 17 | 40 |
| Recipient BMI | ***25*** | 51 | 20 | ***35*** | ***41*** | 29 | 42 | ***48*** | 51 | 28 |
| Peak PRA >80 | ***26*** | 23 | 34 | ***54*** | ***48*** | 51 | 28 | ***35*** | 48 | 32 |
| Immunosuppression class (Anti-proliferative) | ***27*** | 37 | 25 | ***40*** | ***40*** | 35 | 36 | ***45*** | 53 | 41 |
| Candidate hypertension | ***28*** | 26 | 28 | ***53*** | ***34*** | 54 | 34 | ***34*** | 32 | 25 |
| Recipient HCV-positive | ***29*** | 29 | 21 | ***22*** | ***35*** | 52 | 43 | ***13*** | 21 | 10 |
| Cold ischemia time | ***30*** | 30 | 26 | ***34*** | ***24*** | 27 | 26 | ***33*** | 33 | 35 |
| Recipient type 2 diabetes mellitus | ***31*** | 35 | 33 | ***29*** | ***32*** | 32 | 56 | ***19*** | 22 | 21 |
| Donor Diabetes mellitus | ***32*** | 36 | 31 | ***51*** | ***46*** | 56 | 55 | ***32*** | 27 | 45 |
| Immunosuppression class (Calcineurin inhibitors) | ***33*** | 28 | 37 | ***47*** | ***55*** | 47 | 29 | ***39*** | 54 | 44 |
| Donor HCV-positive | ***34*** | 32 | 29 | ***28*** | ***38*** | 34 | 48 | ***17*** | 19 | 23 |
| Previous kidney transplant | ***35*** | 31 | 35 | ***37*** | ***49*** | 46 | 45 | ***52*** | 50 | 33 |
| Immunosuppression class (Corticosteroids) | ***36*** | 47 | 36 | ***33*** | ***25*** | 22 | 46 | ***41*** | 26 | 26 |
| Donor sex (female) | ***37*** | 27 | 42 | ***39*** | ***30*** | 41 | 39 | ***30*** | 30 | 43 |
| Donor cardiac death | ***38*** | 38 | 27 | ***42*** | ***45*** | 30 | 51 | ***36*** | 46 | 39 |
| Employment status (employed) | ***39*** | 34 | 45 | ***14*** | ***27*** | 25 | 30 | ***9*** | 9 | 12 |
| Primary insurance (Other) | ***40*** | 44 | 44 | ***44*** | ***50*** | 48 | 33 | ***37*** | 35 | 29 |
| Any previous transplant | ***41*** | 40 | 40 | ***48*** | ***36*** | 44 | 40 | ***54*** | 37 | 30 |
| Previous transplant | ***42*** | 41 | 41 | ***49*** | ***37*** | 45 | 41 | ***53*** | 38 | 31 |
| Donor alcohol consumption | ***43*** | 46 | 43 | ***36*** | ***54*** | 53 | 47 | ***50*** | 29 | 51 |
| Primary insurance (Medicaid) | ***44*** | 33 | 48 | ***52*** | ***42*** | 55 | 32 | ***51*** | 43 | 47 |
| Recipient sex (female) | ***45*** | 39 | 39 | ***30*** | ***44*** | 36 | 49 | ***26*** | 24 | 55 |
| Recipient type 1 diabetes mellitus | ***46*** | 45 | 47 | ***41*** | ***51*** | 49 | 27 | ***42*** | 49 | 42 |
| Primary insurance (Private) | ***47*** | 43 | 46 | ***27*** | ***28*** | 16 | 44 | ***21*** | 28 | 15 |
| Education level (Some college) | ***48*** | 49 | 52 | ***46*** | ***47*** | 39 | 54 | ***38*** | 55 | 46 |
| Transplant era (After 2010) | ***49*** | 54 | 49 | ***56*** | ***29*** | 28 | 25 | ***56*** | 56 | 56 |
| Donor CMV | ***50*** | 42 | 56 | ***50*** | ***43*** | 43 | 38 | ***43*** | 45 | 34 |
| Primary insurance (Medicare) | ***51*** | 52 | 38 | ***18*** | ***19*** | 17 | 18 | ***18*** | 18 | 24 |
| Education level (College graduate) | ***52*** | 53 | 51 | ***38*** | ***39*** | 42 | 52 | ***55*** | 52 | 48 |
| Donor BMI | ***53*** | 50 | 50 | ***32*** | ***56*** | 37 | 31 | ***27*** | 25 | 19 |
| Glomerulopathy | ***54*** | 48 | 54 | ***23*** | ***31*** | 33 | 37 | ***15*** | 15 | 20 |
| Education level (High school) | ***55*** | 55 | 55 | ***45*** | ***33*** | 40 | 50 | ***46*** | 34 | 27 |
| Terminal serum creatinine | ***56*** | 56 | 53 | ***55*** | ***53*** | 50 | 53 | ***31*** | 44 | 37 |

Variables were ranked based on the VIMP, which was obtained for DCAS and the CR models, using a split-half approach for each outcome. Results between subsets were similar; therefore, we only describe the ranking of variables based on the complete data analyses. For DCAS, the transplant era had the highest VIMP, followed by recipient serum creatinine at discharge and recipient age at transplant, which consistently ranked second and third, respectively. The random forest CR analysis revealed transplant era, delayed graft function (DGF), recipient discharge creatinine, use of immunosuppression drug, recipient age at discharge, and KDRI had the highest VIMPs for allograft failure. Recipient age at transplant, transplantation era, EPTS, use of immunosuppression drug, transplantation between 2001 and 2005, and KDRI were the top six VIMPs for recipient death. EPTS ranked higher in the CR analysis for death than for allograft failure. Finally, recipient employment ranked 9^th^ for mortality and 39^th^ for allograft survival. We tested for interaction effects between the DRP and the top variables in the DCAS and mortality as CR.
